# Visceral Leishmaniasis Mimicking Aggressive Lymphoma

**DOI:** 10.7759/cureus.92843

**Published:** 2025-09-21

**Authors:** Marie Arzel, Cristina Bellini, Dina Milowich, Mitja Nabergoj

**Affiliations:** 1 Internal Medicine, Hôpital Riviera-Chablais, Vaud-Valais, Rennaz, CHE; 2 Infectious Disease, Institut Central des Hôpitaux, Sion, CHE; 3 Pathology, Institut Central des Hôpitaux, Sion, CHE; 4 Hematology, Centre Hospitalier Universitaire Vaudois (CHUV), Lausanne, CHE

**Keywords:** fever of unknown origin, inflammatory syndrome, leishmania, liposomal amphotericin, splenomegaly

## Abstract

We present the case of a 48-year-old immunocompetent male who presented with B-symptoms, inflammatory syndrome, and splenomegaly suggestive of aggressive lymphoma. After a thorough diagnostic workup ruled out lymphoma, visceral leishmaniasis was suspected due to his travel history to a low-endemic area for *Leishmania *spp. Polymerase chain reaction (PCR) confirmed the diagnosis, and the patient was successfully treated with liposomal amphotericin B. This case highlights the importance of considering visceral leishmaniasis in febrile patients with relevant travel histories and the critical need for early diagnosis and treatment.

## Introduction

Visceral leishmaniasis, also known as kala-azar, is a parasitic zoonotic disease caused by *Leishmania* spp. and transmitted by sandflies. Depending on the *Leishmania* spp. and the host immune status, the clinical manifestations are highly variable and can range from cutaneous involvement to potentially fatal visceral disease (kala-azar). Despite 90% of cases being reported in seven countries (India, Bangladesh, Sudan, South Sudan, Ethiopia, and Brazil), the infection is also endemic in Southern Europe (including France, Spain, Portugal, Italy, Albania, Cyprus, Greece, and Malta) [1]. However, it probably remains underdiagnosed in non-endemic regions. In this article, we present a case of a patient who presented with B-symptoms and splenomegaly suggestive of aggressive lymphoma: only after a thoughtful diagnostic workup and anamnesis, visceral leishmaniasis was diagnosed in this patient who travelled in a European endemic area six months before the onset of symptoms.

## Case presentation

A 48-year-old immunocompetent male, previously known for asthma, presented to our hospital's emergency department in South-Western Switzerland with recurrent fever and shivering over the past seven days, accompanied by a persistent cough and unintended weight loss of 15% of his body weight over the preceding weeks. The medical history was otherwise unremarkable, except that the symptoms occurred during the ongoing SARS-CoV-2 pandemic and that the patient had contact with his son, who had scarlet fever. He reported having travelled six months earlier on vacation to Sardinia (southern Italy). He denied any high-risk sexual behavior or intravenous drug use.

At admission, the patient appeared ill and febrile but was hemodynamically stable and had normal findings on a neurologic examination. Physical examination revealed splenomegaly, confirmed by a CT scan showing a spleen of 18 cm, without any lymphadenopathy or other visceral lesions. The laboratory test results (Table 1) revealed mild leucopenia of 2.7 G/L (reference range, 4-10 G/L), including mild neutropenia, mild anemia with a hemoglobin level of 123 g/L (reference range, 133-170 g/L), low platelet count of 127 G/L (reference range, 140-350 × G/L). Further analysis showed hepatitis with elevated alanine aminotransferase (ALT) level of 287 U/L (reference range, <50 U/L), aspartate aminotransferase (AST) of 304 U/L (reference range, <50 U/L), alkaline phosphatase (ALP) of 180 U/L (reference range, <130 U/L), gamma-glutamyltransferase (gGT) of 141 U/L (reference range, <60 U/L) and elevated lactate dehydrogenase (LDH) of 1287 U/L (reference range, <250 U/L). Further work-up showed an inflammatory syndrome with elevated C-reactive protein (CRP) and ferritin (124 mg/L (reference range, <5 mg/L) and 7800 µg/L (reference range, 30-400 µg/L), respectively) and polyclonal hypergammaglobulinemia. Initial microbiological tests were negative, including multiple blood and urine cultures, negative respiratory virus panels, and negative serology for common infections. Due to persistent fever of unknown origin, a transthoracic echocardiographic exam was performed, without signs of endocarditis. 

**Table 1:** Laboratory test results at admission.

Test	Result	Reference Range
White blood cell count	2.7 G/L	4-10 G/L
Neutrophils	1.4 G/L	1.8-7.5 G/L
Hemoglobin	123 g/L	133-170 g/L
Platelets	127 × 10⁹/L	150-350 × 10⁹/L
ALT (alanine aminotransferase)	287 U/L	<50 U/L
ALP (alkaline phosphatase)	180 U/L	<130 U/L
AST (aspartate aminotransferase)	304 U/L	<50 U/L
gGT (gamma-glutamyltransferase)	141 U/L	<60 U/L
LDH (lactate dehydrogenase)	1287 U/L	<250 U/L
CRP	125 mg/L	<5 mg/L
Ferritin	7800 µg/L	30-400 µg/L
Creatinine	89 µmol/L	50-105 µmol/L
LDH (lactate dehydrogenase)	669 U/L	0-249 U/L
Immunoglobulin G (IgG)	17.4 g/L	7-11 g/L
Immunoglobulin A (IgA)	4.2 g/L	0.7-3.5 g/L
Immunoglobulin M (IgM)	3.6 g/L	0.4-2.3 g/L
Immunofixation	Polyclonal	Polyclonal

Due to worsening symptoms, absence of improvement despite broad-spectrum empiric antibiotic therapy, presence of splenomegaly, and associated inflammatory syndrome, an aggressive lymphoproliferative disease was suspected, and an 18F-fluorodeoxyglucose (18-FDG) PET-CT was performed.

**Figure 1:**
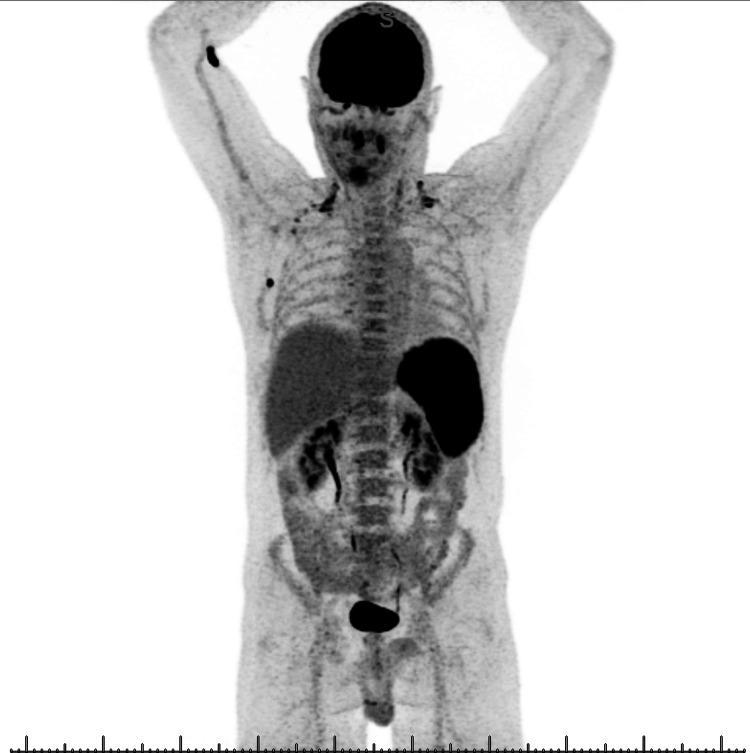
18F-fluorodeoxyglucose (FDG) PET/CT scan shows hypermetabolic splenomegaly and hepatomegaly. Small axillary lymphadenopathies appear reactive. Additionally, diffuse osteomedullary hypermetabolism is noted.

The 18-FDG PET-CT showed hypermetabolic splenomegaly (SUVmax 14.7), homogeneous hepatic and diffuse bone marrow hypermetabolism, as well as sub-centimetric hypermetabolic right axillary lymph nodes, mildly hypermetabolic cervical and right subpectoral lymph nodes, and bilateral nonspecific tonsillar hypermetabolism (Figure 1). A bone marrow biopsy was performed and showed normal findings, while a liver biopsy revealed Kupffer cell hyperplasia and some evidence of phagocytosis without evidence of lymphoma. The absence of monoclonal lymphocytes was also confirmed by flow cytometry on both peripheral blood and bone marrow aspirate.

**Figure 2:**
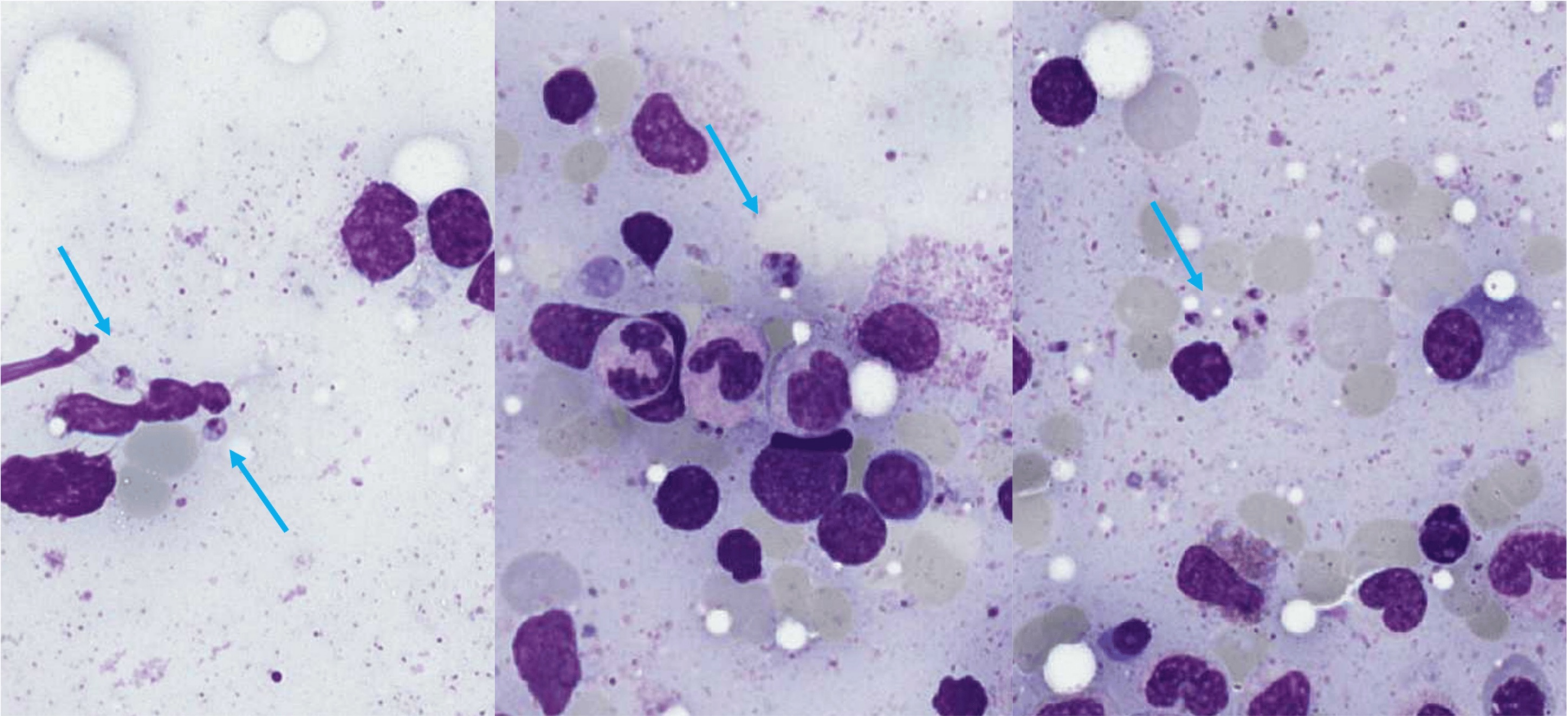
Bone marrow aspirate (May-Grünwald-Giemsa stain, magnification ×100). The arrows indicate the amastigotes.

Once lymphoma was ruled out, based on the patient’s travel history and the clinical and biological data, we suspected visceral leishmaniasis. Blood and bone marrow polymerase chain reactions (PCRs) for *Leishmania* spp. were performed, returning positive. The bone marrow aspiration was reviewed by an expert pathologist, and rare amastigotes (Leishman-Donovan bodies) were noted (Figure 2).

The patient was started on liposomal amphotericin B at a dose of 3 mg/kg for five days, with additional doses administered on days 14 and 21. He rapidly improved clinically and biologically, and a follow-up PCR test performed two months after treatment completion was negative. At the six-month follow-up visit, the patient reported well-being with no recurrence of symptoms.

## Discussion

Leishmaniasis is a parasitic zoonotic disease, and it is primarily transmitted by vector-borne means, predominantly by the female sandfly. Dogs are the primary reservoir of *Leishmania infantum*, the species endemic in the Mediterranean basin, including Sardinia. The incubation period may range from two weeks to eight months, and the clinical presentation can be acute or subacute [2].

Visceral leishmaniasis mainly presents with persistent, intermittent fever and splenomegaly, which may be associated with various degrees of pancytopenia, hepatomegaly, hypergammaglobulinaemia, and weight loss [2]. The differential diagnosis for these findings encompasses a wide range of conditions. Fever can result from infectious diseases, neoplasms, rheumatologic disorders, or other causes. Splenomegaly, especially when associated with pancytopenia, may be seen in infections such as Epstein-Barr virus (EBV) and cytomegalovirus (CMV), as well as in certain rheumatologic diseases and malignancies. Thus, acute and chronic infections-including viral, bacterial, fungal, and mycobacterial etiologies-should be considered, with conditions such as viral hepatitis, malaria, typhoid fever, splenic abscess, and hematologic neoplasms included in the differential. More severe disorders, such as hemophagocytic lymphohistiocytosis (HLH), should be carefully considered in patients presenting with both fever and splenomegaly.

In our patient, the presence of concurrent B symptoms, hypermetabolic splenomegaly on PET-CT, and an associated inflammatory syndrome pointed towards a lymphoproliferative disease. Concurrent cytopenias and elevated liver function tests incited us to organize liver and bone marrow biopsies. However, after ruling out lymphoma and taking into account recent travel to an endemic area, leishmaniasis was clinically suspected, despite the disease's low incidence in the local region [3].

Furthermore, polyclonal hypergammaglobulinemia [4] and severe inflammatory syndrome, which can potentially lead to life-threatening HLH [5], have been described in patients with visceral leishmaniasis. In the context of HLH, parasitic infections are rare but recognized triggers. Leishmaniasis is the most frequent, while other parasites, such as *Plasmodium* spp. and *Toxoplasma* spp., are less commonly implicated [6].

Microscopic detection of *Leishmania* amastigotes in tissue samples is commonly utilized by resource-poor nations. However, the diagnostic gold standard is PCR [7] due to its high sensitivity and specificity, which have been reported to reach up to 100% in one systematic review and meta-analysis [8]. For comparison, direct parasite detection via microscopy has lower sensitivity (54.0-96.4%) and specificity (as low as 46.0%), depending on the quality of the staining reagent and the level of technical expertise [7].

Liposomal amphotericin B remains the first-line treatment for visceral leishmaniasis because of its efficacy and favorable safety profile [9,10]. While liposomal amphotericin B significantly reduces toxicity risks, attention must still be given to potential kidney toxicity, electrolyte imbalances, and infusion reactions. Conventional amphotericin B requires even closer monitoring of renal function and body temperature during administration [11]. Although other treatment options are available, such as miltefosine (which carries teratogenic risks), pentavalent antimonials (whose use is limited by resistance and toxicity), and various combination therapies, liposomal amphotericin B remains the preferred choice when local resources allow. In resource-limited settings, a combination regimen of miltefosine and paromomycin is often favored [2]. Despite cost remaining a major barrier to widespread use, preferential pricing negotiated by the World Health Organization (WHO) and substantial donations from manufacturers have improved accessibility in endemic regions.

Our patient quickly recovered from his symptoms without further recurrence after treatment.

## Conclusions

This case highlights the importance of considering visceral leishmaniasis in febrile patients with relevant travel history and compatible clinical findings. Although this patient initially presented with signs suggestive of an aggressive lymphoma, the diagnosis was reconsidered after multiple biopsies ruled out a lymphoproliferative disorder. A thorough clinical history ultimately led to the identification of leishmaniasis in this patient when his travel history was considered.

Early recognition and treatment are critical to preventing complications associated with this potentially fatal disease. Though uncommon in Central and Northern European countries, such as Switzerland, visceral leishmaniasis should be considered in patients with recent travel to, or who have migrated from, endemic areas, taking into account that the incubation period can be as long as eight months.
